# Evaluation of physicochemical properties of root-end filling materials using conventional and Micro-CT tests

**DOI:** 10.1590/1678-7757-2016-0454

**Published:** 2017

**Authors:** Fernanda Ferrari Esteves TORRES, Roberta BOSSO-MARTELO, Camila Galletti ESPIR, Joni Augusto CIRELLI, Juliane Maria GUERREIRO-TANOMARU, Mario TANOMARU-FILHO

**Affiliations:** 1 Universidade Estadual Paulista, Faculdade de Odontologia, Departamento de Odontologia Restauradora, Araraquara, SP, Brasil.; 2 Universidade Federal da Bahia, Curso de Odontologia, Departamento de Clínica Integrada, Salvador, BA, Brasil.; 3 Universidade Estadual Paulista, Faculdade de Odontologia, Departamento de Diagnóstico e Cirurgia, Araraquara, SP, Brasil.

**Keywords:** Endodontics, Physical properties, Calcium silicate, X-ray microtomography

## Abstract

**Objective:**

To evaluate solubility, dimensional stability, filling ability and volumetric change of root-end filling materials using conventional tests and new Micro-CT-based methods.

**Material and Methods:**

^7^

**Results:**

The results suggested correlated or complementary data between the proposed tests. At 7 days, BIO showed higher solubility and at 30 days, showed higher volumetric change in comparison with MTA (p<0.05). With regard to volumetric change, the tested materials were similar (p>0.05) at 7 days. At 30 days, they presented similar solubility. BIO and MTA showed higher dimensional stability than ZOE (p<0.05). ZOE and BIO showed higher filling ability (p<0.05).

**Conclusions:**

ZOE presented a higher dimensional change, and BIO had greater solubility after 7 days. BIO presented filling ability and dimensional stability, but greater volumetric change than MTA after 30 days. Micro-CT can provide important data on the physicochemical properties of materials complementing conventional tests.

## Introduction

Root-end filling materials must have physicochemical properties in accordance with the standards defined by the American Dental Association (ADA)^1^ and International Organization for Standardization (ISO)^17^. Samples with reduced dimensions have been proposed for some analyses, without changing the accuracy of the method^7^. Solubility is evaluated using standardized samples of the material, which are weighed before and after immersion in distilled water. The resulting loss of mass is expressed as a percentage of the original mass. However, the material may exhibit degradation during storage, or the cement might absorb water. For this reason, the solubility test has limitations^23^, mainly to evaluate hydrophilic materials as calcium silicate-based cements, which has shown a mass increase in the solubility test^21^.

Endodontic cements and root-end filling materials must have dimensional stability to avoid leakage of microorganisms and their toxic products^7^. According to ANSI/ADA Standard No. 57^1^and ISO 6876^17^ specifications, dimensional change is evaluated by means of linear measurement (mm) of standardized specimens before and after immersion in distilled water for 30 days. The main limitation of this test is that dimensional change is based on a linear measurement^6^, and the materials may contract or expand in all directions.

Microcomputed tomography (Micro-CT) is an important tool that may be used to analyze physicochemical properties of endodontic cements, due to its non-destructive characteristic^15^. Cavenago, et al.^8^ (2014) proposed the use of Micro-CT for quantifying the solubility of MTA used as a root-end filling material with different powder-to-water ratios. Silva, et al.^26^ (2017) used Micro-CT to observe dimensional changes and solubility of AH Plus and MTA Fillapex sealers. Micro-CT has also been used to evaluate other properties, such as root canal filling quality^28^ and porosity^3,29^.

Micro-CT may be used to evaluate filling ability and volumetric changes, complementing conventional tests for endodontic materials. The aim of this study was to compare standard tests and new methods to evaluate the physicochemical properties of root-end filling materials using Micro-CT.

## Material and Methods

**Figure 1:**
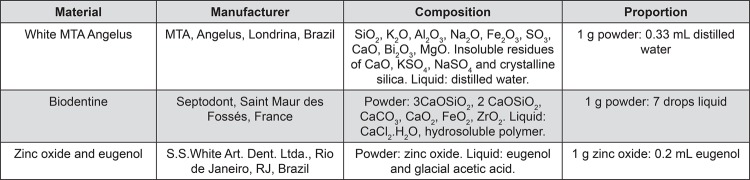
Root-end filling material, manufacturers, and proportions used

MTA Angelus (MTA, Angelus, Londrina, PR, Brazil), Biodentine (BIO, Septodont, Saint Maur des Fossés, Paris, France) and zinc oxide-eugenol cement (ZOE, S.S. White Art. Dent. Ltda., Rio de Janeiro, RJ, Brazil) were used in the proportions described in Figure 1.

### Solubility

Based on a previous study^7^, circular plastic molds measuring 1.5 mm high and 7.75 mm in diameter were placed on a glass plate covered with cellophane film. These molds were filled with each evaluated cement (n=6). A nylon thread was embedded in the fresh cement mixture, and another glass plate covered by cellophane was placed over the mold. This unit was kept at 37°C for 24 hours. The test specimens were removed from the molds and weighed on a precision balance (HM-200, A & D Engineering, Inc., Bradford, MA, USA). Then, they were placed in closed plastic flasks containing 7.5 mL of distilled and deionized water. The specimens were attached to the containers with nylon threads and kept in an oven at 37°C for 7 days. After that period, they were removed from the container, washed in distilled water, and placed in a dehumidifier. The mass was measured before and after the immersion of the samples in distilled water, and every 24 h thereafter, until the mass was stabilized. New samples were made and kept immersed in distilled water for 30 days. The loss of mass was expressed as a percentage of the original mass. In accordance with ISO and ANSI/ADA, the results must not exceed 1.0% of contraction or 0.1% of expansion.

### Dimensional stability

The dimensional stability of the materials was evaluated as previously described^7^. Eight specimens measuring 3.58 mm in height and 3 mm in diameter were fabricated from each material. Their surfaces were polished with 600-grit wet sandpaper. The initial length of each specimen was measured with a digital caliper (Mitutoyo, Tokyo, Japan). The specimens were then stored in flasks containing 2.24 mL distilled water at 37°C for 30 days. Afterwards, they were removed from the flasks, dried with absorbent paper, and their final lengths were determined. The percentage of dimensional change was calculated as follows:

[(L30-L)/L]x100

where L is the initial length of the specimen and L30, the length after 30 days. The test was repeated 3 times. In accordance with ISO and ANSI/ADA, the results must not exceed 1.0% of contraction or 0.1% of expansion.

### Filling ability analysis using Micro-CT

Filling ability of root-end filling materials was analyzed using Micro-CT (Bruker-MicroCT, Kontich, Belgium). Transparent acrylic resin-based models were fabricated using metal molds with cavities measuring 3 mm deep and 1 mm in diameter (n=6). These cavities were filled using contrast solution thickened with bismuth oxide and propylene glycol to evaluate the complete filling of the cavities using Micro-CT. Afterwards, the cavities were cleaned and radiographed using a digital X-ray (Kodak RVG 6100 Digital Radiography System, Marne-la-Vallée, France) to show complete removal of the solution. The cavities were filled with each material using a condenser kit (Ref.: 324501, numbers 2, 3 and 4; Golgran; São Caetano do Sul, SP, Brazil), by a single operator who was previously trained and calibrated. The samples were scanned again. The scanning procedure was performed using 50 kV X-ray tube voltages and 500 μA anode current; aluminum filter of 0.5; isotropic voxel of 18 µm; and an evolution cycle of 360°. Each scanning operation consisted of 721 images in TIF format. These images were used for quantitative analysis of the samples, allowing the total volume of material to be calculated in mm^3^.

**Figure 2:**
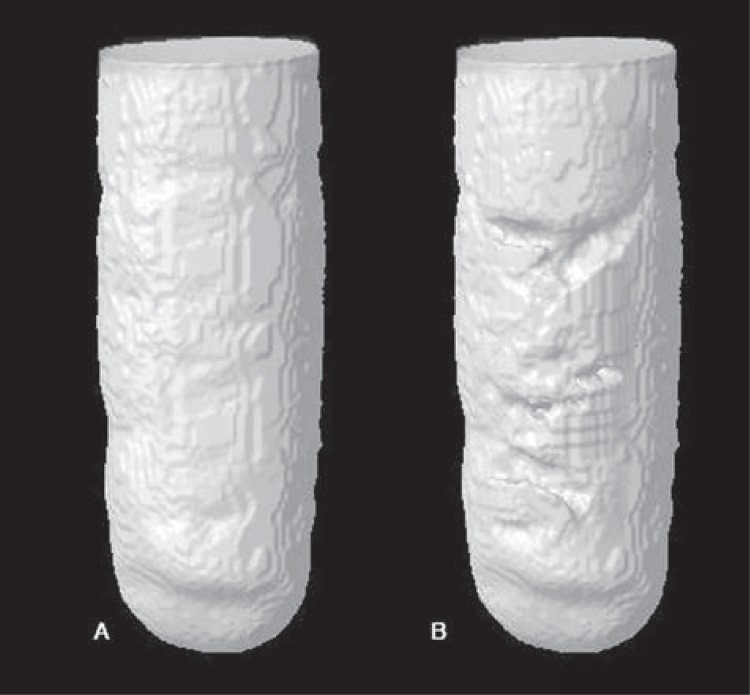
3D model from CTVol software representing the cavity filled with contrast solution (A – 100% of filling) and (B – filled with MTA)

Images were reconstructed using NRecon V1.6.4,7 software (Bruker-MicroCT, Kontich, Belgium). The correction parameters for smoothing, beam hardening and ring artefacts were defined for each material (the parameters for BIO were 0 for smoothing, 80 for beam hardening and 20 for ring artefacts, and, for ZOE and MTA, 0, 50 and 2, respectively). The same parameters were used for the same materials at different periods. The reconstructed images were superposed at the different periods and saved in coronal, sagittal and transaxial planes using the Data Viewer V1.5.2.4 software (Bruker-MicroCT, Kontich, Belgium). The images were analyzed using the CTAn V1.11.8 software (Bruker-MicroCT, Kontich, Belgium). Filling was determined by calculating the difference in volume between the total volume of the cavities, filled with the contrast solution, and the volume obtained after insertion of the different materials. A 3D model of the filled cavities was obtained by using the CTVol V2.0 software (Bruker-MicroCT, Kontich, Belgium) (Figure 2).

### Volumetric change

**Figure 3:**
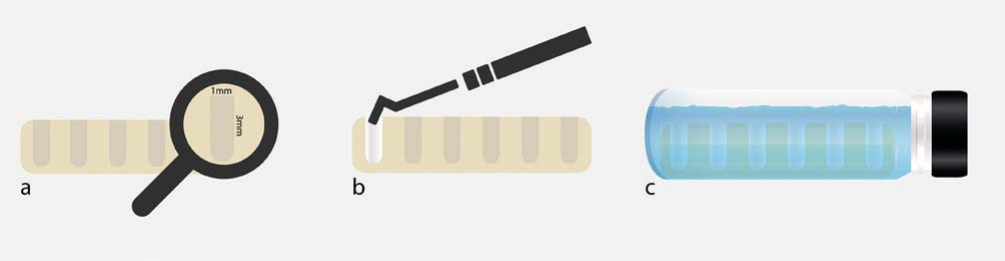
Schematic figure representing the volumetric change assessment [A-Transparent acrylic resin-based models with cavities measuring 3 mm deep and 1 mm in diameter (n=6), B- Cavities filled with each material using a condenser and C- Samples immersed in distilled water between the experimental time intervals (7 and 30 days)]

**Figure 4:**
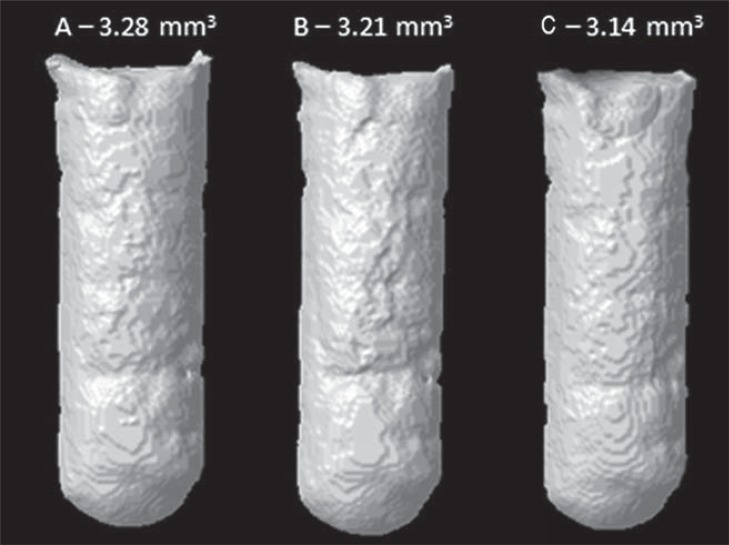
3D model from CTVol software representing the cavity filled with Biodentine in test periods: (A) Initial, (B) after 7 and (C) 30 days of immersion in distilled water. Volumetric filling values in mm3

Cavities in acrylic resin models, measuring 3 mm deep and 1 mm in diameter (n=6) were also prepared to evaluate the volumetric change of the materials by means of Micro-CT. The cavities were filled with each material right after manipulation. The samples were kept in an oven at 37°C and relative humidity for three times the duration of their setting time, and scanned using Micro-CT (Bruker-MicroCT, Kontich, Belgium). The samples were scanned again at 7 and 30 days, and were kept immersed in distilled water between these experimental time intervals. The scanning and analysis parameters were the same as those specified for the filling ability test. The volume filled by the root-end filling materials was calculated at each time interval. A schematic figure of the volumetric change assessment is illustrated in Figure 3. The tridimensional models obtained for the three time intervals (initial, and after 7 and 30 days in water) are represented in Figure 4.

### Statistical analysis

The results obtained for all the tests were submitted to a normality test, and then to the parametric ANOVA statistical test and the Tukey’s multiple comparison test, with 5% significance level.

## Results

BIO and MTA showed higher linear dimensional stability than ZOE cement (p<0.05). At 7 days, BIO showed the greatest solubility (p<0.05), followed by ZOE cement and MTA, which showed the lowest solubility (p<0.05), whereas after 30 days, the materials had similar solubility (p>0.05) (Table 1).

**Table 1:** Mean and standard deviation of results (%) of dimensional change and solubility of root-end filling materials evaluated

Materials/Tests	MTA	BIO	ZOE
Dimensional Change	0.48 (±0.23)^b^	0.31 (±0.23^)b^	-0.99 (±0.15)^a^
Solubility 7 days (mass loss)	-1.62 (±0.46)^c^	6.82 (±0.35)^a^	3.24 (±0.29)^b^
Solubility 30 days (mass loss)	4.66 (±2.26)^a^	6.56 (±1.97)^a^	4.45 (±0.36^)a^

^a,b,c^ Different letters indicate statistically significant difference between experimental groups (p<0.05)

ZOE cement and BIO showed higher volumetric cavity filling ability (p<0.05) than MTA. At 7 days, the materials had similar volumetric change (p>0.05), and, at 30 days, BIO showed greater volumetric change (contraction) than MTA (p<0.05) (Table 2).

**Table 2:** Mean and standard deviation of the results of filling ability (%) and volumetric change (mm3) of root-end filling materials evaluated by Micro-CT

Materials/Tests	MTA	BIO	ZOE
Filling capacity	89.12 (±3.27)^b^	94.22 (±3.93)^a^	96.83 (±3.21)^a^
Volumetric Change 7 days	-1.84 (±0.62)^a^	-1.91 (±0.53)^a^	-1.28 (±0.58)^a^
Volumetric Change 30 days	-2.53 (±0.33)^b^	-4.24 (±0.74)^a^	-3.69 (±1.32)^a,b^

^a,b,c^ Different letters indicate statistically significant difference between experimental groups (p<0.05)

## Discussion

Solubility and dimensional stability were evaluated based on the methodology by Carvalho-Junior, et al.^7^ (2007), with samples of smaller dimensions than those established by ISO 6876/2002^17^ or ANSI/ADA Standard No. 57^1^. According to these standards, dimensional stability is determined by means of linear measurement in a single plane, with an accuracy of evaluation (±1 μm) that may be insufficient for recording small changes^6^. Solubility is evaluated by the difference in mass in grams, between before and after immersion in water, and has limitations that may influence the results^24^. Solubility of a solid material is defined as the amount of a substance dissolved in a solvent. The ISO test measures the elution of a water-soluble material, since the material may present degradation during storage or water absorption^23^. Moreover, the difference in mass may also be insufficient for volumetric behavior analysis, mainly for hydrophilic materials as calcium silicate-based cements, which has shown mass increase in the solubility test^21^. In addition, solubility is evaluated by the ISO standards after a period of 24 hours, but longer analysis periods may be used^9,13,27^, the most widely used time interval being 7 days^12,13,30^.

The limitations of the conventional tests proposed by ISO and ANSI/ADA made it necessary to search for methodologies that provide data to complement conventional tests. The methodologies proposed in this study can complement ISO’s methodology, allowing the appropriate choice of materials, and increasing the clinical treatment success.

The use of microcomputed tomography (Micro-CT) in this study enabled a tridimensional volumetric analysis (in mm^3^) of the materials, complementing the conventional test of dimensional stability analysis. Furthermore, analysis after the time intervals of immersion in distilled water complemented the evaluation of solubility of the materials, and enabled better understanding of the dimensional behavior and solubility of the materials for longer periods. Considering that the evaluated materials were immersed after the setting time, as recommended in the ISO and ANSI/ADA tests, this procedure may be an alternative to evaluate hydrophilic materials. Micro-CT is a non-invasive technique, widely used in Endodontics^15^. The images acquired by using Micro-CT allowed the same specimen to be used at different time intervals of analysis, with a high level of detail^2^. The protocols developed for using this tool to evaluate material filling ability allowed tridimensional analysis of the filling percentage. This analysis showed that ZOE cement and BIO presented greater filling ability, and ZOE cement presented the highest percentage filling rates of the three, in agreement with the study by Shetty, et al.^25^ (2015).

MTA has been reported to promote proper apical sealing^4,13^ and BIO has a better consistency for manipulation^4^, possibly contributing to the better filling properties observed in this study. Koubi, et al.^20^ (2012) evaluated the marginal integrity of restorations by using BIO, and related that the reduced size of the calcium silicate cement particles and small expansion of the material could contribute to its greater filling ability.

MTA presented the lowest filling ability of the three materials, probably because its consistency makes it difficult to manipulate and insert into the root-end cavity^4,22^. Regarding dimensional stability, MTA showed an expansion of 0.48 (±0.23)%, corroborating the study by Islam, et al.^18^ (2006). This may be related to its hydration reaction^10^. The solubilization of the material observed after 30 days most likely occurred because the bismuth oxide used as a radiopacifier increased the porosity of the material, thus also increasing its solubility, as reported in previous studies^5,11,16^. However, in the volumetric change test after 7 and 30 days, MTA showed some alteration. This may be related to its Portland cement-based composition, containing an insoluble silica matrix that maintains its integrity even when in contact with water^14^. This water sorption in hydrophilic materials may difficult the solubility evaluation^26^. Moreover, in the volumetric change test only part of MTA is placed in contact with the aqueous solution, as in a clinical application, while in the solubility test the whole specimen is placed in contact with a large amount of distilled water^14^.

At 7 days, solubility was greater for BIO, and, after 30 days, all the materials had similar solubility. BIO showed higher percentage values, which may have occurred because of the polycarboxylate in its composition; this superplasticizer is used to facilitate the manipulation and insertion of this cement. Conversely, it has a surfactant effect that may have increased the solubility of the material^12^. These results agree with those of previous studies, which have shown higher solubility values for BIO^12,19,27^, suggesting a correlation between this property and the greater volumetric loss observed in this study after 30 days. This result corroborates Cavenago, et al.^8^ (2014), who associated volumetric change with MTA solubility. Dawood, et al.^12^ (2014) investigated the physical properties of Biodentine and MTA Angelus, and observed greater solubility for Biodentine after 7 days. Singh, et al.^27^ (2015) compared the solubility of Biodentine and MTA at the time intervals of 24 hours, 3, 10, 30 and 60 days, and demonstrated that Biodentine showed greater solubility at the time intervals of 30 and 60 days. Kaup, et al.^19^ (2015) evaluated the solubility of Biodentine and MTA ProRoot and found that Biodentine showed greater solubility after 28 days, indicating a mass loss of 4.610 (±1.402)%.

## Conclusions

Zinc oxide-eugenol cement presented higher dimensional change, and Biodentine, greater solubility after 7 days. Biodentine presented filling ability, but greater volumetric change than MTA after 30 days. The tests proposed using Micro-CT provided tridimensional data and complemented the tests recommended by the ISO and ANSI/ADA standards. Micro-CT may be a standardized and reproducible method of analysis.
